# Added value of PHI in predicting lymph node invasion in prostate cancer: an external validation

**DOI:** 10.1186/s12894-026-02243-w

**Published:** 2026-07-09

**Authors:** Jiong Fu, Xiaozhou Zhou, Yang Liu, Hao Deng, Huawang Lyu, Zhiwen Chen

**Affiliations:** Department of Urology, The First Affiliated Hospital of Army Medical University, Chongqing, China

**Keywords:** Prostate cancer, Lymph node invasion, Nomogram, Prostate Health Index (PHI), External validation, Extended pelvic lymph node dissection

## Abstract

**Purpose:**

To determine the predictive performance of the Briganti 2017 and MSKCC nomograms with the addition of Prostate Health Index (PHI) in a Chinese cohort of prostate cancer patients undergoing robot-assisted radical prostatectomy and extended pelvic lymph node dissection.

**Methods:**

This single-center retrospective study included 473 prostate cancer patients who underwent robot-assisted radical prostatectomy with extended pelvic lymph node dissection from Dec. 2021 to Dec. 2025. The two nomograms were validated, and PHI was incorporated as a continuous variable. Model performance was assessed using AUC, calibration plots, and decision curve analysis, with the DeLong test for AUC comparison. This study was registered in the Medical Research Registration and Filing Information System of the National Health Security Information Platform of China (Registration No.: MR-50-25-036796, retrospectively registered).

**Results:**

Lymph node invasion (pN1) was confirmed in 26.0% (123/473) of the cohort. The original Briganti 2017 and MSKCC nomograms had AUCs of 0.69 (95% CI: 0.61–0.79) and 0.71 (95% CI: 0.62–0.78) with satisfactory calibration. PHI incorporation significantly improved discrimination, raising their AUCs to 0.787 (absolute improvement 0.093, *P* < 0.01) and 0.782 (absolute improvement 0.077, *P* < 0.01), respectively. Decision curve analysis showed PHI expanded the threshold probability range of positive net benefit from 10% to 30% to 10%-40% for both models.

**Conclusion:**

The Briganti 2017 and MSKCC nomograms showed acceptable predictive performance for lymph node invasion in Chinese prostate cancer patients. PHI enhances their discriminatory ability and clinical utility, supporting its use as a valuable preoperative biomarker for pN1 in prostate cancer.

**Supplementary Information:**

The online version contains supplementary material available at 10.1186/s12894-026-02243-w.

## Background

Prostate cancer (PCa) is a major global health burden and one of the most common malignancies in men. The accurate preoperative prediction of pelvic lymph node invasion (LNI) is crucial for tailoring individual treatment strategies, particularly in determining the necessity of an extended pelvic lymph node dissection (ePLND), which balances staging accuracy against potential surgical morbidity [1].

Currently, the Memorial Sloan Kettering Cancer Center (MSKCC) nomogram and the Briganti series nomograms are the foremost internationally recognized tools for predicting lymph node invasion (LNI). The MSKCC model is widely used in North America, while the Briganti nomograms are specifically recommended by the European Association of Urology (EAU) guidelines [2]. A significant limitation, however, is that these models were developed and validated predominantly in European and American populations. Their performance and generalizability in Asian, particularly Chinese, men remain insufficiently established, with robust external validation data notably lacking for the MSKCC nomogram in this demographic [3]. Concurrently, the Prostate Health Index (PHI), a blood-based biomarker combining total PSA, free PSA, and [-2]proPSA, has demonstrated superior specificity for clinically significant prostate cancer and a strong association with pathological features of tumor aggressiveness [4–7]. Nevertheless, its specific role and incremental value for predicting LNI, especially when integrated into established nomograms, have not been thoroughly investigated, and relevant data from Chinese patients are scarce.

Despite the established value of these nomograms in Western populations, their performance in Chinese patients remains uncertain. Furthermore, whether the addition of PHI can improve their predictive accuracy for lymph node invasion has not been investigated. Therefore, the objective of this study was to determine the predictive performance of the Briganti 2017 and MSKCC nomograms with the addition of PHI in a contemporary Chinese cohort undergoing robot-assisted radical prostatectomy and extended pelvic lymph node dissection.

## Research methods

### Study design and population

This single-center retrospective study included 473 patients who underwent robot-assisted radical prostatectomy (RARP) with extended pelvic lymph node dissection (ePLND) at our institution between Dec. 2021 and Dec. 2025. Per institutional protocol, ePLND was performed in all patients meeting EAU intermediate- or high-risk classification criteria (based on PSA level, biopsy ISUP Grade Group, and clinical T stage), as well as in EAU low-risk patients with suspicious lymph nodes on preoperative imaging. All ePLND procedures followed a standardized template as described below. Exclusion criteria were: (1) receipt of neoadjuvant endocrine therapy or radiotherapy; (2) missing key clinical variables (e.g., initial PSA or biopsy pathology). The study was approved by the institutional ethics board, and informed consent was waived due to its retrospective design.

### Data collection

Two established nomograms were externally validated: the Briganti 2017 and Memorial Sloan Kettering Cancer Center (MSKCC) nomograms. The predictor variables for each nomogram were collected as defined by their original publications. Additional clinical data, including age, Prostate Health Index (PHI), and radiological staging, were also recorded. All PSA and PHI measurements were obtained prior to diagnostic prostate biopsy. Preoperative imaging workup included multiparametric magnetic resonance imaging (mpMRI) of the prostate and pelvis in all patients, except for those with contraindications such as metallic implants or cardiac pacemakers, in whom transrectal ultrasound was used instead. Whole-body bone scintigraphy was performed routinely. Computed tomography (CT) and prostate-specific membrane antigen positron emission tomography/computed tomography (PSMA PET/CT) were not part of the standard preoperative assessment. Prostate biopsy was performed via a systematic 12-core transrectal approach without formal MRI-targeted cores.

### Surgical and pathological procedures

All RARP and ePLND procedures were performed by a consistent senior surgical team using the Da Vinci Surgical System via a transperitoneal approach. The ePLND template covered the distal and superior aspects of the common iliac vessel bifurcation, extending along the external iliac vessels and including nodes lateral to the internal iliac vessels and within the obturator fossa. Pathological examination was conducted by uropathologists per International Society of Urological Pathology (ISUP) standards [8, 9]. Lymph nodes, together with the surrounding adipose tissue, were embedded en bloc for pathological examination. The final lymph node yield incorporated all grossly palpable nodules as well as those identified only upon microscopic examination.

### Statistical analysis

#### Evaluation of the original predictor-set models

Because patient-level original nomogram scores were not retrievable, we refitted multivariable logistic regression models in our cohort using the same preoperative predictor sets as the Briganti 2017 and MSKCC nomograms. These are termed the original predictor-set models. Discrimination was assessed with the area under the receiver operating characteristic curve (AUC). Calibration was evaluated using calibration plots with LOWESS smoothing, calibration intercept, calibration slope, and Brier score. Decision curve analysis (DCA) was performed across clinically relevant threshold probabilities [10].

#### Construction and validation of PHI-extended models

The Prostate Health Index (PHI) was added as a continuous predictor to each original model, yielding PHI‑extended logistic regression models. All coefficients were re-estimated in the PHI complete-case subset. AUCs were compared with those of the corresponding original predictor-set models using the DeLong test [11]. Calibration was assessed as described above. Net reclassification improvement (NRI) and integrated discrimination improvement (IDI) were calculated to further quantify the incremental value of PHI.

To correct for overoptimism, bootstrap internal validation with 1,000 resamples was performed, and optimism‑corrected AUC, Brier score, and calibration slope are reported. Model equations and coefficients are provided in Supplementary Tables S1–S2.

#### Clinical impact analysis

To translate model performance into clinical decision-making, threshold-specific clinical impact analyses were performed at selected risk thresholds relevant to ePLND. For each threshold, we calculated the number of patients classified as requiring ePLND, the number of true-positive pN1 patients, the number of false-positive pN0 patients, the number of missed pN1 patients, and the number of potentially avoidable unnecessary ePLNDs.

All analyses were performed in R (version 4.4.2) with two‑sided *P* < 0.05 considered significant.

## Results

### Perioperative baseline characteristics

A total of 473 patients who underwent RARP with ePLND were included in the analysis. The baseline clinical and pathological characteristics are summarized in Table 1. The median age was 69 years (IQR 64–73), with a median initial PSA of 18.5 ng/mL (IQR 10.4–41.9) and a median PHI of 66.4 (IQR 44.5–124.2). Regarding preoperative imaging, mpMRI of the prostate and pelvis was performed in 449 patients (94.9%), transrectal ultrasound was performed in 78 patients (16.5%), whole-body bone scintigraphy was performed in 457 patients (96.6%), and PSMA PET/CT was performed selectively in 39 patients (8.2%). CT was not part of the standard preoperative assessment. Prostate biopsy was performed via a systematic 12-core transrectal approach in 391 patients (82.7%) or a transperineal approach in 82 patients (17.3%). Preoperative biopsies showed ISUP Grade Groups 4–5 in 72.1% of patients, which was consistent with the final pathology in 71.3% of postoperative specimens. The majority of patients (93.5%) were classified as having intermediate- or high-risk disease according to the EAU risk stratification. Most patients (69.8%) had clinical or radiological T-stage ≤T2. Postoperatively, positive surgical margins were identified in 56 patients (11.8%), and pN1 were confirmed in 123 patients (26.0%). The median number of lymph nodes removed was 19 (IQR 15–25).

**Table 1:** Perioperative characteristics of patients who underwent robot-assisted radical prostatectomy and extended pelvic lymph node dissection

	All included patients (*n* = 473)
Age at surgery (yr), median (IQR)	69(64–73)
^a^Initial PSA value (ng/ml), median (IQR)	18.5(10.4–41.9)
^d^Initial PHI value, median (IQR)	66.4(44.5-124.2)
Preoperative imaging, n (%)	
mpMRI	449 (94.9)
Transrectal ultrasound	78 (16.5)
Whole-body bone scintigraphy	457 (96.6)
PSMA PET/CT	39 (8.2)
Biopsy route, n (%)	
Transrectal	391 (82.7)
Transperineal	82 (17.3)
^a^Biopsy grade group according to ISUP, n (%)	
1	22(4.6)
2	34(7.2)
3	67(14.2)
4	138(29.2)
5	212(42.8)
EAU risk group, n (%)	
Low risk	31(6.5)
Intermediate risk	71(15.1)
High risk	371(78.4)
^b^Highest-grade positive cores, median (IQR)	33.3 (16.7–58.3)
^b^Lower-grade positive cores, median (IQR)	16.7 (8.3–33.3)
^c^Number of positive cores, median (IQR)	4 (1–7)
^c^Number of negative cores, median (IQR)	6 (4–9)
^a^Radiological T stage, n (%)	
Negative (rT1c)/organ-confined disease (rT2)	330(69.8)
Extracapsular extension (rT3a)	62(13.1)
≥Seminal vesicle invasion (rT3b)	81(17.1)
Final histopathological grade according to ISUP, n (%)	
1	12(2.5)
2	44(9.3)
3	79(16.7)
4	90(19.1)
5	247(52.2)
Surgical margin status, n (%)	
Negative	417(88.2)
Positive	56(11.8)
Number of removed lymph nodes, median (IQR)	19(15–25)

*^a^ Variables used in the original Briganti 2017 and MSKCC nomograms.*^b^ Variable specific to the Briganti 2017 nomogram.*^c^ Variable specific to the MSKCC nomogram.*^d^ Variable added for the PHI-extended models

### External validation of the original nomograms

**Fig. 1:**
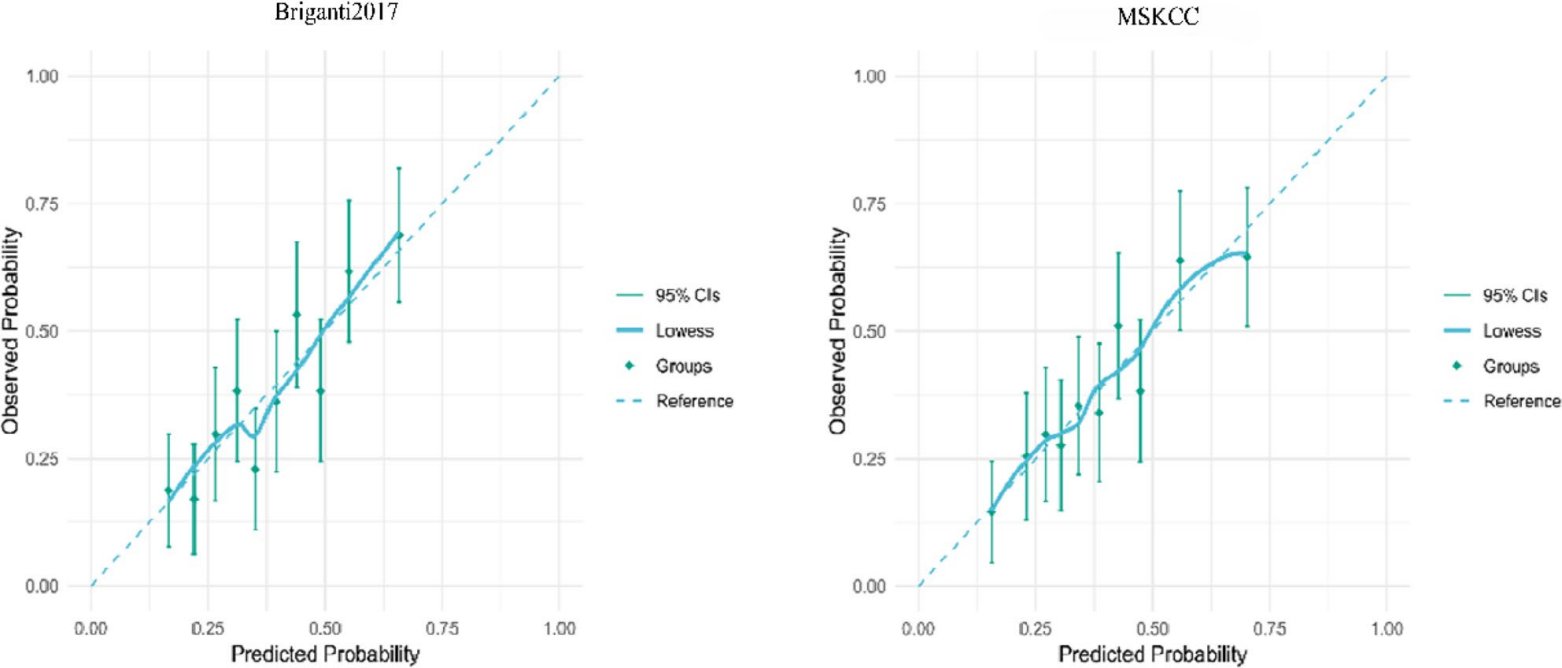
Calibration plots of the original predictor-set models for the Briganti 2017 (left) and MSKCC (right) nomograms.* The diagonal dashed line represents perfect calibration. The solid blue line represents the LOWESS smoothed observed-versus-predicted curve. The green dots indicate the observed probabilities for each group. The vertical green lines represent the 95% confidence intervals

Calibration plots for the Briganti 2017 and MSKCC nomograms are illustrated in Fig. 1. Both models exhibited robust calibration performance: the locally weighted scatterplot smoothing (LOWESS) curves closely aligned with the diagonal reference line, especially when the predicted probability of LNI was below 40%. Throughout the full range of predicted risk, the LOWESS curves of both nomograms showed a steady upward trend with increasing predicted LNI risk, reflecting strong consistency between predicted probabilities and actual clinical outcomes. The 95% confidence intervals effectively enveloped the LOWESS curves, confirming the stability of the predictive performance across different risk strata (Fig. 1). For discriminatory ability, the Briganti 2017 nomogram had an AUC of 0.69 (95% CI: 0.61–0.79), and the MSKCC nomogram had an AUC of 0.71 (95% CI: 0.62–0.78).

**Fig. 2:**
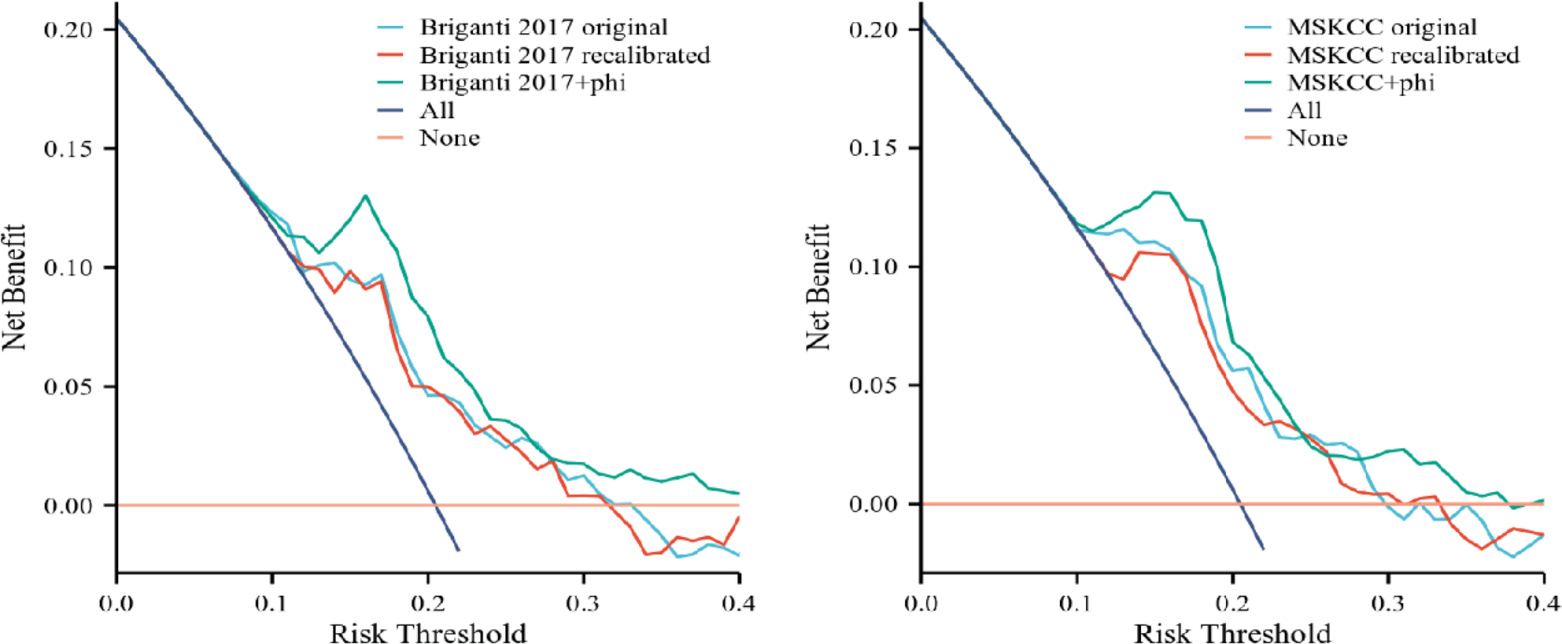
Decision curve analysis of the original predictor-set models and PHI-extended models for the Briganti 2017 (left) and MSKCC (right) nomograms. *The x-axis represents the threshold probability for defining high-risk LNI, and the y-axis represents the net benefit. The horizontal orange line represents the “treat none” strategy, and the diagonal dark blue line represents the “treat all” strategy. The light blue curve represents the original predictor-set model, the red curve represents the recalibrated model, and the green curve represents the PHI-extended model

Decision curve analysis revealed that the original and recalibrated Briganti 2017 nomograms both provided a positive net benefit at LNI threshold probabilities of 10% − 30%. For the MSKCC nomogram, the original version provided a positive net benefit at 10%–30% threshold probabilities; recalibration expanded this range to 15%–35%, improving its clinical applicability (Fig. 2).

### Model performance after incorporating PHI

**Fig. 3:**
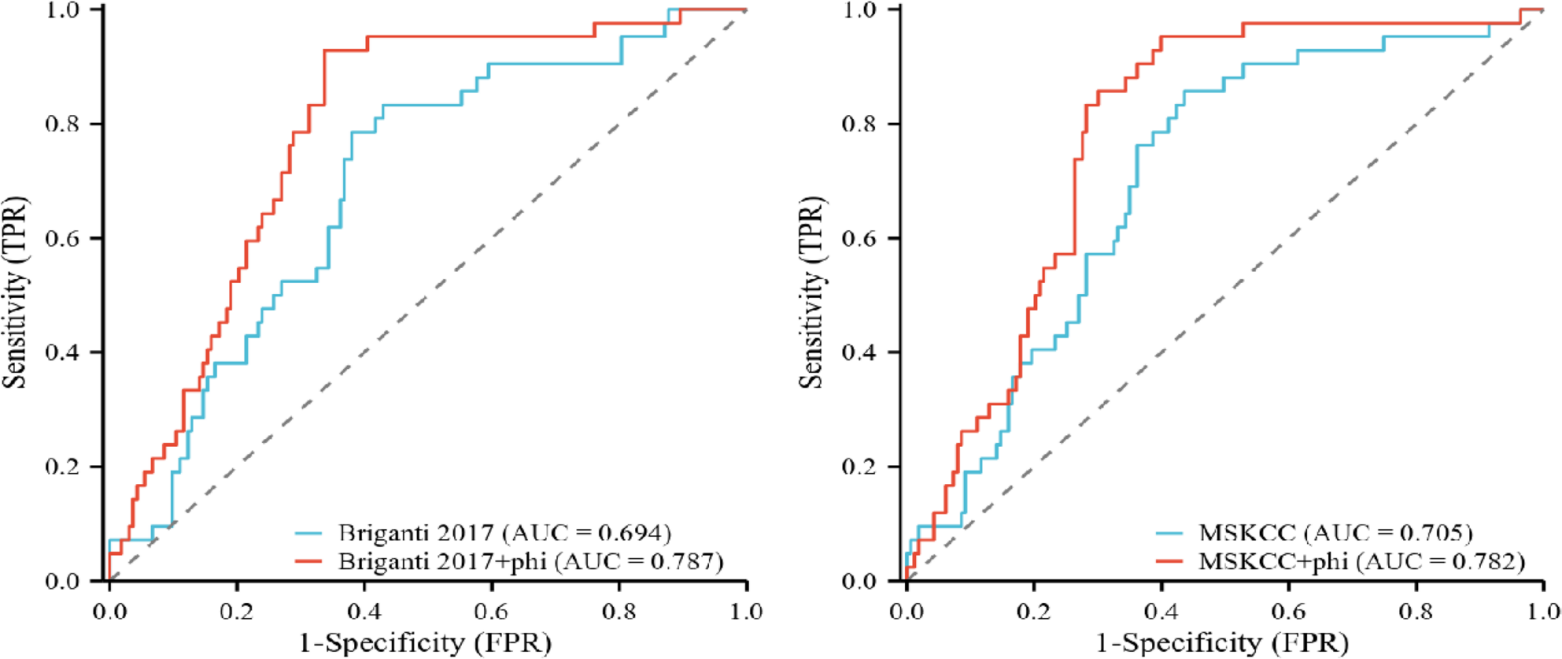
ROC curves of the original predictor-set model and PHI-extended model for the Briganti 2017 (left) and MSKCC (right) nomograms. *The blue curve represents the original predictor-set model, and the red curve represents the PHI-extended model. AUC=area under the curve

Integration of the Prostate Health Index (PHI) as a continuous predictor into the original Briganti 2017 and MSKCC nomograms resulted in a substantial enhancement of predictive performance. Specifically, the AUC of the Briganti 2017 model increased to 0.79 (95% CI, 0.72–0.86), representing an absolute improvement of 0.09 compared to the original model. For the MSKCC nomogram, the AUC rose to 0.78 (95% CI, 0.71–0.85), with an absolute improvement of 0.07. Both enhancements in discriminatory ability reached statistical significance (*P* < 0.01 for both comparisons) (Fig. 3).

Decision curve analysis further validated the clinical value of PHI integration: the original Briganti 2017 nomogram offered a positive net benefit at threshold probabilities of 10%–30%, while this beneficial range extended to 10%–40% after PHI addition. Similarly, the MSKCC nomogram’s positive net benefit range expanded from 10% to 30% (original model) to 10%–40% following PHI incorporation, indicating broader clinical applicability across different risk thresholds. (Fig. 2) Both PHI-extended models demonstrated satisfactory calibration, and bootstrap internal validation confirmed the stability of the performance estimates (Supplementary Tables S3 and S4). The addition of PHI also improved risk reclassification. For the Briganti 2017 predictor set, the continuous NRI was 0.640, with 57.1% of patients with pN1 correctly reclassified upward and 74.8% of patients without pN1 correctly reclassified downward. The IDI was 0.037, indicating a significant improvement in integrated discrimination. For the MSKCC predictor set, the continuous NRI was 0.628, with similar patterns of reclassification (57.1% of pN1 patients upward; 74.2% of pN0 patients downward), and the IDI was 0.034. Category-based NRI, using clinically relevant risk thresholds (10%–40%), was 0.394 for the Briganti 2017 predictor set and 0.388 for the MSKCC predictor set. Detailed NRI and IDI results are provided in Supplementary Table S7. Threshold-based clinical impact analysis further demonstrated the practical value of PHI-enhanced models. At the commonly used 15% threshold, the Briganti 2017 + PHI model reduced unnecessary ePLND recommendations by 12 patients while missing 3 fewer pN1 cases compared with the original predictor-set model. At the 20% threshold, the MSKCC + PHI model reduced false-positive recommendations by 12 patients and missed 5 fewer pN1 cases. Across the clinically relevant range of 10%–20%, the PHI-enhanced models consistently reduced both false positives and missed pN1 cases, indicating that PHI integration can help refine existing cutoff-based strategies by simultaneously improving patient selection for ePLND and reducing overtreatment. Detailed results are provided in Supplementary Table S8.

## Discussion

This is the first external validation of the Briganti 2017 and MSKCC nomograms for predicting lymph node invasion (LNI) in a Chinese prostate cancer cohort. Additionally, we assessed the incremental clinical value of integrating the Prostate Health Index (PHI) into these prediction tools. Our key findings are summarized as follows: First, both nomograms exhibited acceptable and comparable discriminatory performance in our cohort (AUC: 0.69 for Briganti 2017 and 0.71 for MSKCC, respectively), with calibration curves demonstrating good agreement between predicted probabilities and observed LNI outcomes. Second, incorporating PHI as a continuous predictor significantly improved the discriminatory ability of both models, with AUC increases of approximately 0.08–0.09. Decision curve analysis (DCA) further supported the clinical utility of the enhanced models, showing an expanded range of threshold probabilities (10%–40%) over which the models provided greater net clinical benefit for clinical decision-making.

The AUC values observed in our cohort were slightly lower than those reported in the original development studies and subsequent Western validation cohorts [12] which may be attributable to several factors. First, our cohort had a higher proportion of high-risk patients (93.5%) and a substantially higher LNI rate (26.0%) than the original Briganti 2017 development cohort (~ 15%) [13], this enrichment of higher-risk patients in modern surgical cohorts likely reflects three trends: the widespread adoption of mpMRI and selective PSMA PET/CT; the increasing use of active surveillance for low-risk disease; and the expansion of surgical indications to older and higher-grade patients owing to improvements in robotic-assisted techniques. Such spectrum shifts have been noted in other external validation studies [14] and may partly explain the lower AUCs observed in contemporary cohorts.Second, differences in PSA screening strategies, indications for lymph node dissection, and pathological processing standards between Chinese and Western clinical practice may further contribute to the discrepancy [15, 16] .Finally, potential biological differences between Asian and Western populations could also influence model performance. Consistent with our results, Fukagawa et al. [3] reported a comparable AUC of 0.71 in their Japanese external validation of the Briganti 2019 nomogram. As a result, the findings from this and other contemporary validation studies should be interpreted within the context of high-risk surgical populations and may not be generalizable to lower-risk men, such as those undergoing active surveillance or those with low pre-test probabilities of LNI.Despite the modest reduction in discriminatory ability, the good calibration of both models supports their clinical applicability in Chinese patients, though further optimization may be warranted to enhance predictive accuracy for this population.

Our findings confirm that PHI confers significant incremental value for predicting LNI in prostate cancer. Although prior studies have linked PHI to prostate cancer aggressiveness [5–7], its specific role in LNI prediction remained inadequately explored. To our knowledge, this study is the first to integrate PHI into established LNI prediction nomograms, demonstrating that PHI not only enhances model discriminatory ability but also expands the range of threshold probabilities with meaningful clinical utility. The biological rationale underlying PHI’s predictive value is compelling: compared to total PSA, PHI exhibits higher specificity for malignant prostatic tissue. Its key component, [-2]proPSA, is predominantly expressed by malignant prostate epithelial cells, and its levels correlate positively with high Gleason scores and tumor aggressiveness [4, 5]. Elevated PHI levels may thus indicate the presence of highly aggressive tumor clones with enhanced migratory and invasive potential, which could foster a microenvironment conducive to lymphangiogenesis and subsequent nodal dissemination [17, 18]. Consequently, increased serum PHI levels may reflect a higher likelihood of micrometastatic events, making it a valuable complementary predictor to conventional nomograms that rely primarily on anatomic and morphological parameters. It should be acknowledged, however, that most patients in our cohort underwent systematic biopsy with prior knowledge of MRI findings, and operators typically directed additional cores toward MRI-suspicious regions. This practice differs from the purely systematic biopsy setting of the original Briganti 2017 and MSKCC models (neither of which incorporated MRI-derived variables) and from the software-registration-guided MRI-targeted biopsy procedure required by the Briganti 2019 model [19]. Consequently, the observed incremental value of PHI may partly reflect limitations of the older predictor sets, rather than an independent predictive gain beyond contemporary MRI-era models.

Furthermore, PHI holds distinct practical advantages over PSMA-PET/CT in making precision prostate cancer staging more accessible. While PSMA-PET/CT improves nomogram performance when added as a predictor [14], its widespread deployment is constrained by high costs, the need for specialized equipment and radiopharmaceuticals, and reliance on expert radiological interpretation [20]. In contrast, PHI requires only a routine blood sample, entails no expensive instrumentation, and provides objective, standardized results. This accessibility positions PHI as a complementary, scalable tool—not a competitor to PSMA-PET—particularly suited to resource-limited settings such as central and western China, where PSMA-PET/CT availability remains scarce. In these contexts, PHI-enhanced nomograms may serve as a practical first-line refinement, identifying patients who would benefit most from subsequent advanced imaging or more extensive surgical staging. In the present study, both the “Briganti 2017 + PHI” and “MSKCC + PHI” models demonstrated a positive net benefit across a threshold probability range of 10%–40%, supporting their utility in guiding clinical decisions on ePLND. These enhanced models are thus valuable for surgical planning in intermediate- or high-risk patients and provide an evidence base for developing regionally adapted prediction tools for the Chinese population.

Finally, sentinel lymph node biopsy (SLNB) represents an emerging alternative to ePLND for nodal staging in localized prostate cancer. Recent randomized and prospective studies have demonstrated that indocyanine green (ICG)-guided SLNB can achieve accurate nodal staging—with per-patient accuracy rates approaching 98% and negative predictive values as high as 97%—while substantially reducing postoperative complications compared with standard ePLND (32% vs. 70%) [21, 22]. Moreover, ICG guidance during ePLND has been shown to increase the yield of retrieved lymph nodes and improve the detection of metastatic disease, with corresponding improvements in biochemical recurrence-free survival [23]. Standardized pelvic lymph node mapping, as recently developed by the Pelvic Rosetta Classification project, may further refine the anatomical framework for SLNB and image-guided nodal dissection [24]. In the context of our findings, whether preoperative risk models incorporating PHI could help identify candidates most suitable for SLNB rather than standard ePLND represents an intriguing direction for future investigation.

Our study has several acknowledged limitations. First, its single-center, retrospective design and highly selected cohort—93.5% of patients were intermediate- or high-risk, with a lymph node invasion rate of 26.0% (*n* = 123)—may introduce spectrum bias and limit the generalizability of the findings to broader or lower-risk populations. Although the absolute number of node-positive cases was not negligible, it may still compromise the stability of the model estimates. Second, despite standardized surgical and pathological protocols, all patients were treated at a single tertiary institution, which may introduce selection bias or institutional assessment bias. Third, we were unable to evaluate the more recent Briganti 2019 nomogram, which requires software-registration-guided MRI-targeted biopsy [19] and was therefore not applicable to our study population. Moreover, most patients in our cohort underwent systematic biopsy with prior knowledge of MRI findings, and operators typically directed additional cores toward MRI-suspicious regions—a practice that differs from the purely systematic biopsy setting of the original models. These factors together suggest that the observed incremental value of PHI may partly reflect limitations of the older predictor sets, rather than an independent predictive gain beyond contemporary MRI-era models. Finally, although bootstrap internal validation was performed to correct for overoptimism, the PHI-extended models have not been validated in an independent external cohort, and their generalizability remains to be confirmed.

## Conclusion

In this Chinese cohort, the Briganti 2017 and MSKCC nomograms demonstrated acceptable predictive performance for lymph node invasion. PHI, as a cost-effective and readily accessible biomarker, significantly enhanced both the discriminatory ability and clinical utility of these models. These findings support the potential role of PHI as a valuable adjunct for preoperative risk stratification.

## Supplementary Information

Supplementary Material 1.

Supplementary Material 2.

Supplementary Material 3.

Supplementary Material 4.

Supplementary Material 5.

Supplementary Material 6.

Supplementary Material 7.

Supplementary Material 8.

Supplementary Material 9.

## Data Availability

All data generated or analyzed during this study are included in the present article. Further enquiries regarding the data can be directed to the corresponding author (Xiaozhou Zhou, E-mail: zhouxz@tmmu.edu.cn).

## Abbreviations

AUC: Area under the curve
CT: Computed tomography
DCA: Decision curve analysis
EAU: European Association of Urology
ePLND: Extended pelvic lymph node dissection
GG: Grade Group
ICG: Indocyanine green
IDI: Integrated discrimination improvement
ISUP: International Society of Urological Pathology
LNI: Lymph node invasion
mpMRI: Multiparametric magnetic resonance imaging
MRI: Magnetic resonance imaging
MSKCC: Memorial Sloan Kettering Cancer Center
NRI: Net reclassification improvement
PCa: Prostate cancer
PET: Positron emission tomography
PHI: Prostate Health Index
PSA: Prostate-specific antigen
PSMA: Prostate-specific membrane antigen
RARP: Robot-assisted radical prostatectomy
ROC: Receiver operating characteristic
SLNB: Sentinel lymph node biopsy
TRUS: Transrectal ultrasound
