# Supplementary material for: Added value of PHI in predicting lymph node invasion in prostate cancer: an external validation
Source: BMC Urol. 2026 Jul 9;26:159. doi: 10.1186/s12894-026-02243-w (PMC13348157; doi:10.1186/s12894-026-02243-w)
Supplement: Supplementary file 9 — Supplementary Material 9. [file 12894_2026_2243_MOESM9_ESM.docx]

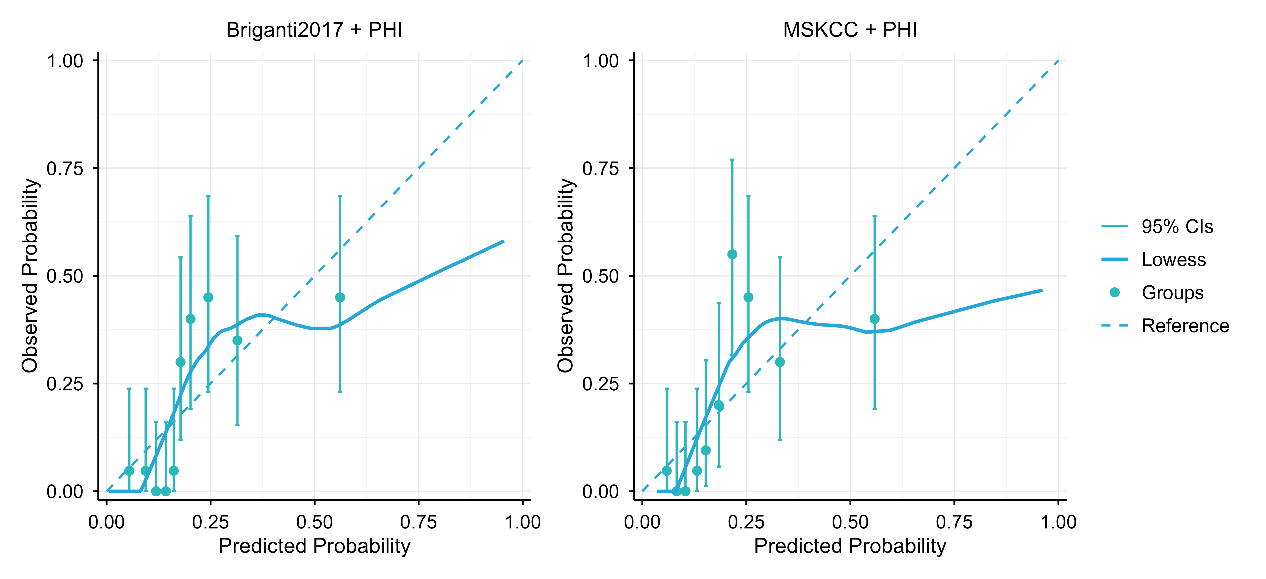


**Supplementary Figure S1***. Calibration plots of the PHI-extended models. (Left) Briganti 2017 + PHI model. (Right) MSKCC + PHI model*. **The diagonal dashed line represents perfect calibration. The solid blue line represents the LOWESS smoothed observed-versus-predicted curve. The green dots indicate the observed probabilities for each group. The vertical green lines represent the 95% confidence intervals. Both PHI-extended models showed satisfactory calibration, with curves closely following the diagonal reference line.*
